# Synthesis of Sucrose-HDI Cooligomers: New Polyols for Novel Polyurethane Networks

**DOI:** 10.3390/ijms23031444

**Published:** 2022-01-27

**Authors:** Csilla Lakatos, Marcell Árpád Kordován, Katalin Czifrák, Lajos Nagy, Bence Vadkerti, Lajos Daróczi, Miklós Zsuga, Sándor Kéki

**Affiliations:** 1 Department of Applied Chemistry, Faculty of Science and Technology, University of Debrecen, Egyetem tér 1, H-4032 Debrecen, Hungary; lakatoscsilla@science.unideb.hu (C.L.); kordovan.marci@science.unideb.hu (M.Á.K.); czifrak.katalin@science.unideb.hu (K.C.); nagy.lajos@science.unideb.hu (L.N.); vadkerti.bence@science.unideb.hu (B.V.); zsuga.miklos@science.unideb.hu (M.Z.); 2 Doctoral School of Chemistry, University of Debrecen, Egyetem tér 1, H-4032 Debrecen, Hungary; 3 Department of Solid State Physics, Faculty of Science and Technology, University of Debrecen, Bem tér 18/b, H-4026 Debrecen, Hungary; daroczi.lajos@science.unideb.hu

**Keywords:** sucrose, 1,6-hexamethylene diisocyanate, cooligomer, polyol, poly(ε-caprolactone), chemical and physical characterization

## Abstract

Sucrose-1,6-hexamethylene diisocyanate (HDI) cooligomers were synthesized and used as new polyols for poly(ε-caprolactone) (PCL)-based polyurethanes. The polyaddition reaction of sucrose and HDI was monitored by MALDI-TOF MS. It was found that by selecting appropriate reaction conditions, mostly linear oligomer chains containing 16 sucrose units could be obtained. For the synthesis of polyurethane networks, prepolymers were prepared by the reaction of poly(ε-caprolactone) (PCL, 10 kg/mol) with HDI or 4,4′-methylene diphenyl diisocyanate (MDI) and were reacted with sucrose-HDI cooligomers. The so-obtained sucrose-containing polyurethanes were characterized by means of attenuated total reflectance Fourier-transform infrared spectroscopy (ATR-FT IR), swelling, mechanical (uniaxial tensile tests) and differential scanning calorimetry (DSC).

## 1. Introduction

Polyurethanes (PUs) are a versatile family of polymers. The incorporation of various constituents [1,2] into the polymer chains allows one to tailor their properties for certain applications. Such properties include, e.g., mechanical and thermomechanical properties as well as biocompatibility and biodegradability. Thus, PUs based on polyesters such as polycaprolactones have gained considerable interest as biodegradable polymers [3,4,5]. In addition, semi-crystalline polycaprolactones (PCL) provide good flexibility [6] and often act as switching components in shape memory polymers (SMPs). 

Recently, carbohydrates, due to their biocompatibility, easy accessibility and low cost, are frequently applied in polymer syntheses as monomers [7,8,9,10] and crosslinkers [11,12]. Their favorable biological properties enable them to be used in many fields of medicine [13,14], ranging from drug delivery, implants and wound healing to cancer therapy [15,16,17,18,19] and as injectable SMPU embolic agents [20,21]. Furthermore, these polymers are also expected to degrade in the human body or in nature [22]. Moreover, incorporation of carbohydrates of natural origin into polymer chains, due to the presence of several reactive functional groups (OHs), can lead to uncontrolled synthesis [23,24,25]. Sucrose, as a cheap and easily available carbohydrate, is used for the preparation of various polymers including polyesters, polyurethanes and resins. However, these polymers are rigid and difficult to handle [26]. 

Therefore, the combination of the sucrose-1,6-hexamethylene diisocyanate (HDI) copolymers with the soft polycaprolactone segment is expected to compensate for the stiffening effect of the sugar units. It is to be noted, however, that in order to obtain linear sucrose-HDI chains, two OH groups of sucrose should preferentially react with the isocyanate groups of HDI. According to our previous investigations, there are three reactive primary and five less reactive secondary OH groups in the sucrose molecule [27]. Furthermore, it was found that the two primary OH groups in the positions of 6 and 6′ are the most reactive ones [27]. In this article, we report the synthesis and characterization of long sucrose-HDI polymer chains (containing up to 16 sucrose units) built into the polycaprolactone-based polyurethane networks. The polyurethanes we produced may be suitable for the development of a new type of scaffold after the biocompatibility has been verified.

## 2. Results and Discussion

### 2.1. Synthesis of the Sucrose-HDI Cooligomer

Recently, we investigated the reaction of unprotected sucrose hydroxyl groups with phenyl isocyanate and found that the sterically and electronically less hindered primary hydroxyl groups (in the positions of 6 and 6′) are more reactive than the third one in the position of 1′ [28]. Based on this finding, we assumed that long linear chains could be synthesized using HDI and MDI. It should be noted, however, that in addition to the sterically hindered primary hydroxyl group of position 1′, the secondary ones may also react with 1,6-hexamethylene diisocyanate (HDI) or 4,4′-methylene diphenyl diisocyanate (MDI) to form branches and/or networks. Thus, to obtain long oligomer chains, sucrose and HDI or MDI were reacted in a 1:1 mole ratio in DMSO at 80 °C under an argon atmosphere. The reactions were followed by MALDI-TOF MS to determine the optimal reaction time to avoid the undesirable side reactions, i.e., branching. The MALDI-TOF MS spectra of reaction mixture quenched with methanol were recorded in the linear mode.

**Figure 1:**
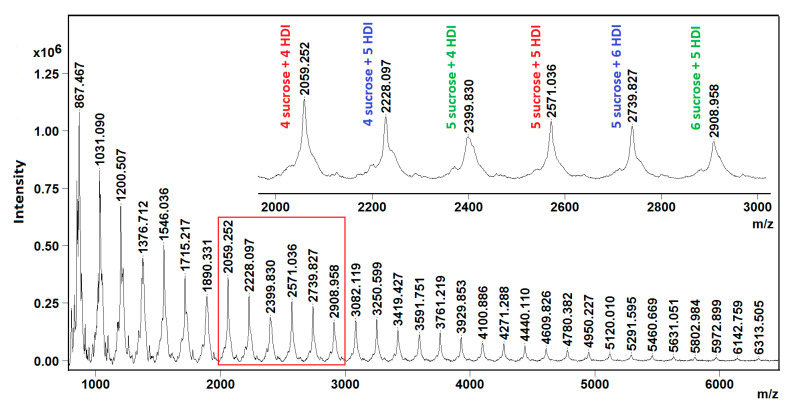
MALDI-TOF MS spectrum of the products of sucrose-HDI reaction after 4 h at 80 °C recorded in the linear mode. The applied mole ratio of the reactants was 1:1. Three series were observed (sucrose-HDI series (red), HDI-sucrose-HDI series (blue), sucrose-HDI-sucrose (green)) as depicted in the inset.

Figure 1 represents the MALDI-TOF MS spectra recorded between 800 and 6300 *m*/*z*. As seen in Figure 1, the formation of oligomers up to 6300 *m*/*z*, corresponding to oligomer chains with 16 sucrose units, can be observed. Furthermore, as Figure 1 inset shows, there are three different series repeated regularly throughout the mass spectrum. These series were assigned to be oligomers with one (i.e., sucrose-HDI series, n_HDI_ = n_sucrose_, where n_HDI_ = number of HDI and n_sucrose_ = number of sucrose units) and two HDI end-groups (i.e., HDI-sucrose-HDI series, n_HDI_ = n_sucrose_ + 1) and the third series was identified as sucrose-HDI-sucrose oligomer (n_HDI_ + 1 = n_sucrose_), as shown in the inset. In the case of longer sucrose-HDI chains, there is an increasing opportunity for the formation of branches by involving the secondary OH groups in the urethane-forming reaction. Hence, at reaction times longer than four hours, the appearance of branches and additional side reactions becomes significant. During the reaction monitored for 6 days (144 h), the molecular weight of the oligomer consisting of sucrose and HDI units increased to 8000 m/z (see Figure S1).

### 2.2. Syntheses of Sucrose-HDI Cooligomer Containing SUPUs Networks

**Scheme 1:**
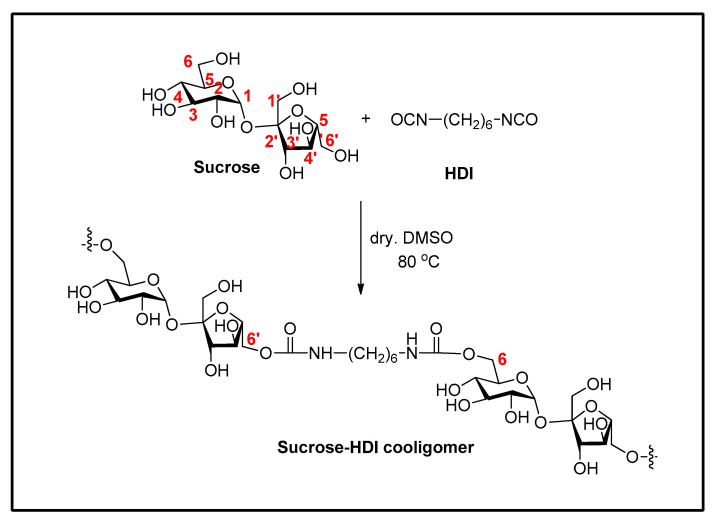
Synthesis of sucrose-HDI cooligomers.

**Scheme 2:**
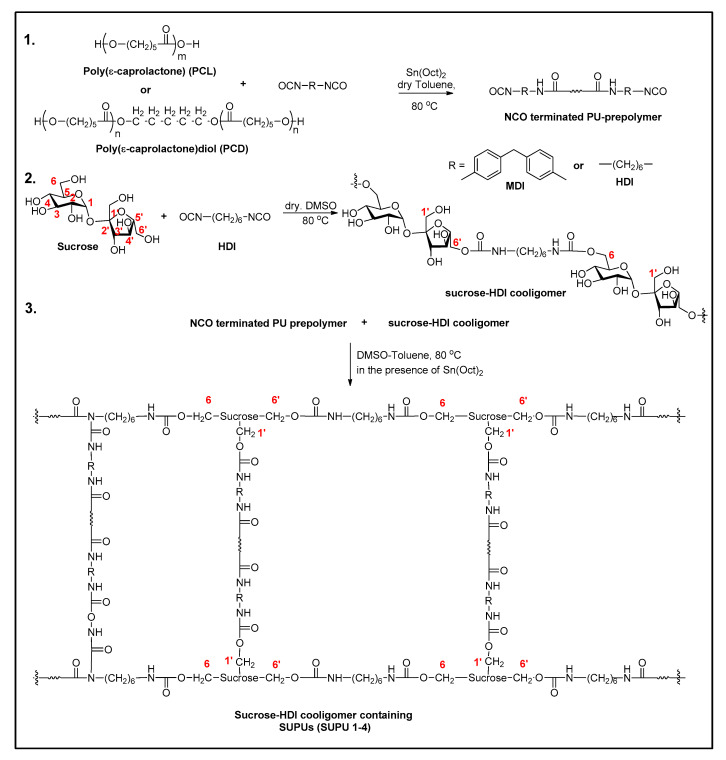
Syntheses of polyurethane elastomers containing sucrose-HDI cooligomers.

Our further goal was to synthesize sucrose containing polyurethanes (SUPUs). Based on the results of MS analysis, 4 h reaction time was chosen for the preparation of sucrose-HDI cooligomers (Scheme 1). During the preparation of the sucrose-HDI cooligomer, HDI or MDI was added in one portion to the sucrose containing DMSO solution. The mole ratio of sucrose to HDI in the reaction mixture was 1:1. To synthesize SUPU networks, the DMSO solution of the “in situ” formed sucrose-HDI cooligomer was added to the toluene solution of PCL-based prepolymer. The crosslinks were formed by polyaddition reaction between the NCO terminated prepolymer and the remaining OH groups of sucrose units. The applied compositions of the reaction mixtures are listed in the Materials and Methods section. The steps of the synthesis are summarized in Scheme 2.

### 2.3. Infrared Spectroscopy (FT-IR)

**Figure 2:**
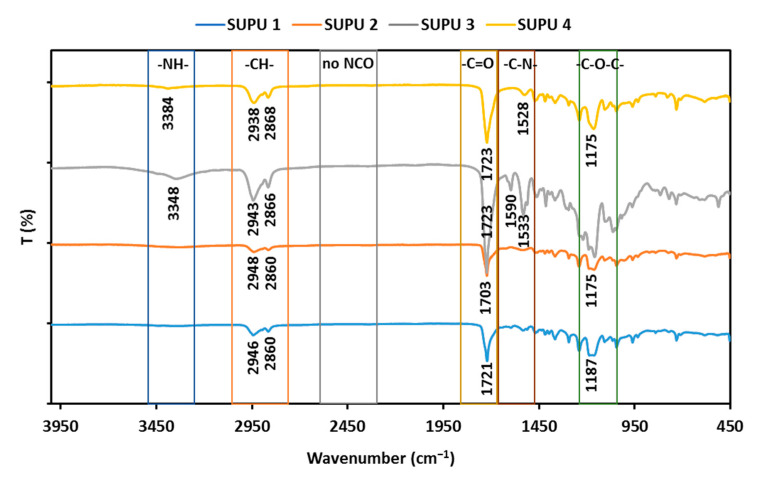
ATR-FT IR spectra of the synthesized SUPU samples in the range of 450−4000 cm^−1^. The marked areas represent the characteristic bands.

**Figure 3:**
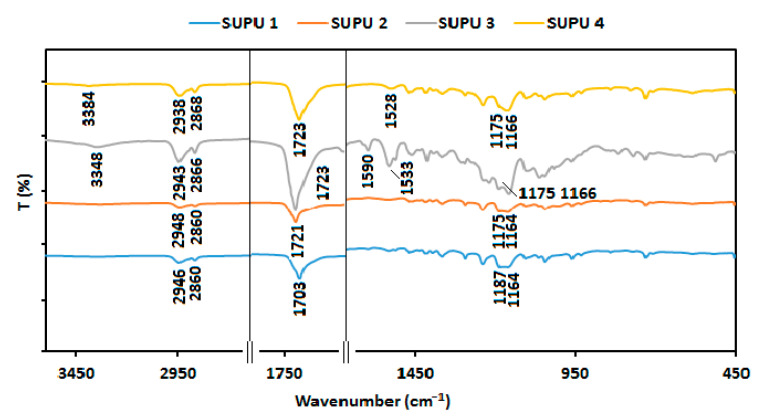
The detailed IR spectra of SUPUs 1–4.

Some structural information can be obtained by ATR-FT IR spectroscopy of sucrose cooligomer containing polyurethanes (SUPUs 1–4), as depicted in Figure 2 and Figure 3. The IR spectra of sucrose-HDI cooligomer and prepolymer of SUPU 4 are shown in Figures S2 and S3 in the supporting information.

The zoomed IR spectra are presented in Figure 3.

The absence of stretching vibration around 2260 cm^−1^ (isocyanate group vibrations) indicates the complete transformation of NCO groups. The broad peak found in the range of 3384–3348 cm^−1^ is due to the -N-H stretch vibration in the urethane bonds and the -O-H vibrations in the sucrose units.

The peaks between 2948 and 2938 cm^−1^ and 2868 and 2860 cm^−1^ indicate a large number of aliphatic CH_2_ groups (C-H stretch vibration).

The relatively broad -C=O vibration that appeared between 1723 and 1703 cm^−1^ suggests that the polyurethane contains H-bonded and non H-bonded C=O groups belonging to the ester and urethane bonds.

Moreover, the presence of urethane bonds is also indicated by the peaks around 1590 and 1533 cm^−1^ (amide I and II peaks).

The peak around 1180 cm^−1^ can be interpreted as the -C-O-C- vibration in the polycaprolactone chains and corresponding to cyclic structures of disaccharide for sucrose.

### 2.4. Swelling Properties of SUPUs Networks

In order to support the crosslinked structure of SUPUs (SUPU 1–4), swelling tests were performed. In the swelling experiment, SUPU samples of well-defined weight were swollen in toluene. Since no sample could be dissolved in toluene (except for SUPU 4, see later), the incorporation of the sucrose-HDI cooligomer into the SUPU networks resulted in a crosslinked structure. As it turns out from the data of Table 1, swelling data show that sample SUPU 3 has the highest gel content (G = 96.2%). On the other hand, sample SUPU 1 reveals the highest degree of swelling (Q = 15.7), while dissolution of sample SUPU 4 was observed, indicating the presence of very loose crosslinks (or the absence of them). The crosslink densities determined from toluene swelling experiments spanned from 1.2 × 10^−4^ to 1.7 × 10^−3^ mol/cm^3^. This finding provides a clear support for the network formation.

### 2.5. Morphology of SUPUs by SEM

**Figure 4:**
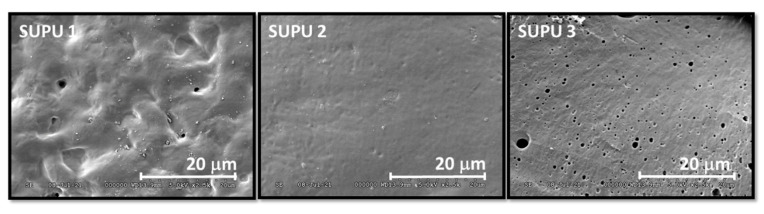
SEM recordings regarding SUPUs 1–3 for the visualization of morphology.

In order to visualize the morphology of SUPUs 1–3, SEM images were taken. As illustrated in Figure 4, similar microstructures were formed during syntheses and curing. In addition, no significant phase separation was observed. Based on these results, it can be surmised that sucrose containing polyurethane networks have a nearly homogeneous microstructure.

### 2.6. Mechanical Properties of SUPUs

**Figure 5:**
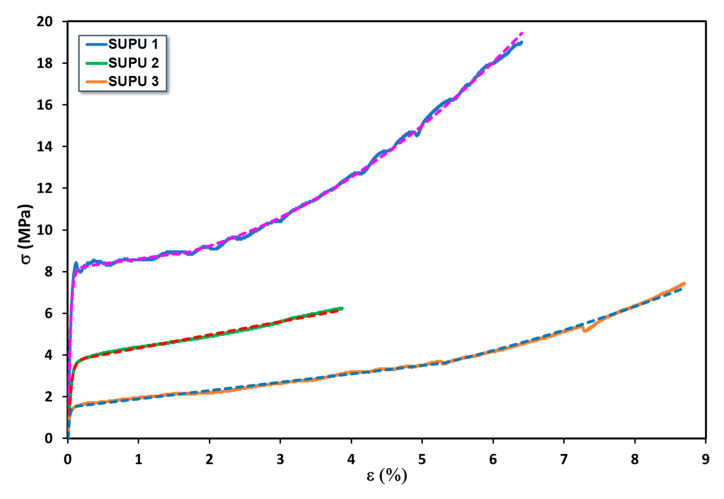
The stress–strain (σ–ε) curves for samples SUPU 1–3. The solid and the dashed lines stand for the experimental and the fitted curves (Equations (6) and (7)), respectively. The fitted parameters for the sample SUPU 1 are [(dε/dt)a_1_]^−1^ = 30.08, a_2_ = 0.47 MPa, (dε/dt)a_3_ = 8.15 MPa, α = 0.28 and ε_L_ = 1.5, for SUPU 2: [(dε/dt)a_1_]^−1^ = 23.91, a_2_ = 0.63 MPa, (dε/dt)a_3_ = 3.70 MPa, α = 0 and for SUPU 3: [(dε/dt)a_1_]^−1^ = 48.22, a_2_ = 0.40 MPa, (dε/dt)a_3_ = 1.50 MPa, α = 0.76 and ε_L_ = 5.3.

The mechanical properties of samples SUPU 1–4 were studied using the uniaxial tensile test. The results of these investigations are compiled in Table 2 and Figure 5.

As seen in Table 2, both the type of diisocyanate and the polycaprolactone segment influence the resulting mechanical properties of the SUPU samples. For example, samples synthesized with MDI (SUPU 1 and SUPU 2) possess higher elastic moduli and stress at break than those prepared with HDI (SUPU 3 and SUPU 4), indicating the chain stiffening effect of the aromatic isocyanate MDI. The exception to that is the higher elastic modulus of SUPU 4 as compared to that of SUPU 3, which may be due to the initially present higher physical crosslink density in sample SUPU 4. Furthermore, it can also be concluded that the chemically crosslinked samples (i.e., SUPU 1, SUPU 2 and SUPU 3) with longer polycaprolactone chains demonstrate a higher elastic modulus and stress at break (SUPU 1 and SUPU 3) than their version containing shorter PCL segments (SUPU 3). This finding may be ascribed to the higher crystallinity fraction of PCL in these chemically crosslinked samples. On the other hand, samples SUPU 1, SUPU 2 and SUPU 3, due to their crosslinked structure, show reasonable elongation at break values (from 405% to 784%), while the linear (or very loosely crosslinked) sample SUPU 4 reveals only a small value (13%), which can be attributed to the “fast” decomposition of the physical crosslinks upon elongation.

In order to describe the stress versus relative strain curves, we used the Standard Linear Solid (SLS) model (Equation (1)) extended with an additional equation (Equation (2)) for the strain-hardening effect.

(1) $$\frac{d\sigma }{d\varepsilon }=\;{\left(\frac{d\varepsilon }{\mathrm{dt}}a_{1}\right)}^{-1}\left[a_{2}\left(\varepsilon \right){\varepsilon +a}_{3}\frac{d\varepsilon }{\mathrm{dt}}\;-\;\sigma \right]$$

(2) $$a_{2\;}\left(\varepsilon \right)=\;a_{2}+\alpha \;{{(\varepsilon -\varepsilon }_{L})}^{\beta }\;(\mathrm{if}\;\varepsilon \;>\;\varepsilon_{L})$$

where σ and ε are the stress and relative strain, respectively, a_1_, a_2_, a_3_ and α are the parameters to be determined, while ε_L_ is the “critical” strain at which strain-hardening occurs.

In our calculations β = 1 was applied, and Equations (1) and (2) were numerically integrated and fitted to the experimental stress–strain curves to obtain the target parameters. Interestingly, Equations (1) and (2) were also applied successfully for the description of various polyurethane systems [12,29,30]. As seen in Figure 5, the fitted curves match all the experimental stress–strain curves, indicating the utility and capability of the extended SLS model for such investigations.

### 2.7. Thermal Analysis of SUPUs

**Figure 6:**
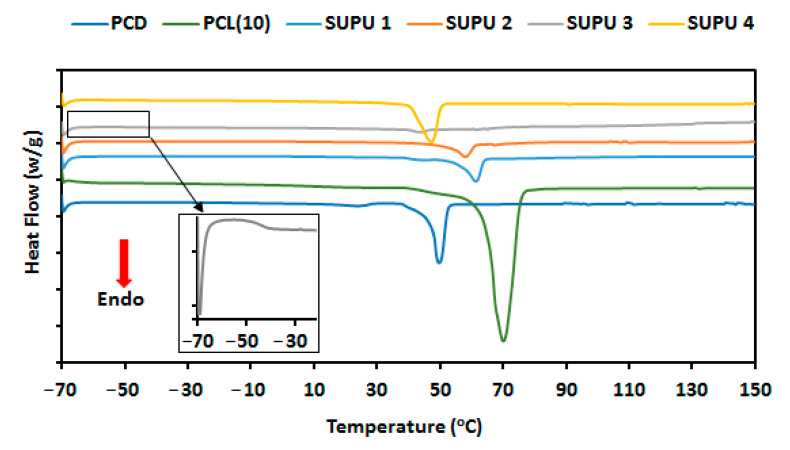
DSC traces for the polycaprolactone and samples SUPUs 1–4. The peaks between 53 and 64 °C refer to the melting of the PCL, while those between 38 and 47 °C belong to the PCD units.

The DSC traces are shown in Figure 6 and the data obtained from DSC curves and the calculated crystallinity values are presented in Table 3.

According to the data of Table 3 and Figure 6 T_g_ of the pure PCL and PCD did not occur due to their low values (both being below −70 °C) [31]. Moreover, T_g_ of PCD in SUPU 3 appeared at −44 °C. Thus, this finding indicates a considerable increase in the T_g_ of PCD in sample SUPU 3, which is likely due to the considerable restriction of chain movements exerted by the crosslinks (see Table 1). Furthermore, both the T_m_ and the degree of crystallinity (C_r_) of PCL decreased with respect to those of the pure PCL. This observation is in line with the crosslinked structure of samples SUPU 1 and SUPU 2, i.e., crosslinks restrict the crystallization of PCL segments. Similar behavior can be observed in connection with the T_m_ and C_r_ of sample SUPU 3, but in that case, the crystalline fraction of PCD considerably reduced (60% vs. 8%). On the other hand, the T_m_ and C_r_ of sample SUPU 4 do not change significantly with respect to the pure PCD. This finding is in good agreement with the linear or the very loosely crosslinked structure of SUPU 4. In addition, it can also be seen from the data in Table 3 that incorporation of MDI or HDI into the chains in the case of samples SUPU 1 and SUPU 2 does not have a significant effect either on the T_m_ or on the C_r_ values.

## 3. Materials and Methods

### 3.1. Materials

For the cooligomer synthesis, D(+)-sucrose (puriss, Ph. Eur. 6.) from Reanal (Budapest, Hungary) was dried in a vacuum oven at 40 °C for overnight. 1,6-hexamethylene diisocyanate (HDI) (reagent grades, 98%), 4,4′-methylene diphenyl diisocyanate (MDI) (reagent grades, 98%), poly(ε-caprolactonediol) (PCD) M_n_ = 2 kg/mol and poly(ε-caprolactone) (PCL) M_n_ = 10 kg/mol from Sigma-Aldrich (Darmstadt, Germany) were used without further purification. Tin(II) 2-ethylhexanoate (M = 405.12 g/mol, 92.5–100%), dimethyl sulfoxide (DMSO) (ACS reagent, 99.9%) and toluene (reagent grade, 99.7%), distilled over P_2_O_5_ and stored on sodium wire were purchased from Sigma-Aldrich (Darmstadt, Germany).

### 3.2. Synthesis

#### 3.2.1. Monitoring the Sucrose-HDI Cooligomer Formation

Dried sucrose (0.25 g, 0.73 mmol) was dissolved in 4 mL anhydrous DMSO at 80 °C. After complete dissolution of sucrose, HDI (0.123 g, 0.18 μL, 0.73 mmol) was added to the solution in one portion under an argon atmosphere. The temperature of the reaction mixture was kept at 80 °C and from time to time an aliquot of 50 μL was withdrawn and analyzed by means of MALDI-TOF MS.

#### 3.2.2. Preparation of the Sucrose-HDI Cooligomer Solution

A given amount of dried sucrose (see in Table 4) was dissolved in anhydrous DMSO (8 mL) in a three-necked flask equipped with CaCl_2_ tube, condenser and gas inlet. After dissolution of sucrose, 1 equivalent HDI was added in one portion to the reaction mixture stirred and kept at 80 °C under argon atmosphere for 4 h.

#### 3.2.3. Preparation of the PU-Prepolymers

For the synthesis of sucrose-HDI cooligomer-containing polyurethanes, poly(ε-caprolactone) PCL (M_n_ = 10 kg/mol) or poly(ε-caprolactonediol) PCD (M_n_ = 2 kg/mol) was dissolved in 20 mL dry hot toluene placed in a three-necked flask equipped with a condenser and a CaCl_2_ tube. Then, 2 equivalent of 1,6-hexamethylene diisocyanate (HDI) or 4,4′-methylene diphenyl diisocyanate (MDI) was added to the solution in the presence of 3 drops from a Pasteur pipette (~2 mol %) of the tin(II)2-ethylhexanoate catalyst. The reaction mixture was continuously stirred at 80 °C for 2 h.

#### 3.2.4. Preparation of the Sucrose-HDI Cooligomer-Containing (SUPU) PUs

For the preparation of sucrose-HDI cooligomer-containing PU polymers, the solutions of sucrose-HDI cooligomer and PU-prepolymer were combined and stirred for a further 2 h at 80 °C. After 2 h reaction time, the reaction mixture was poured into Teflon plate and left to dry at 40–50 °C until constant weight. The products obtained were colorless or yellowish elastic films (yield: 98–99%).

The compositions of samples SUPU 1–4 are compiled in Table 5.

### 3.3. Characterization

#### 3.3.1. Matrix-Assisted Laser Desorption///Ionization Time-of-Flight Mass Spectrometry (MALDI-TOF)

For the matrix-assisted laser desorption///ionization time-of-flight mass spectrometry (MALDI-TOF MS) measurements, a Bruker Autoflex Speed mass spectrometer equipped with a time-of-flight///time-of-flight (TOF///TOF) mass analyzer (Bruker Daltoniks, Bremen, Germany) was used. The ions were detected in the positive ion mode using a 19 kV acceleration voltage. To achieve appropriate resolution and mass accuracy, the reflectron mode with 21 and 9.55 kV voltages were employed. For the desorption of ions, a solid phase laser (355 nm, ≥100 μJ/pulse) operating at 500 Hz was applied and 5000 shots were added. The MALDI-TOF MS spectra were externally calibrated with polyethylene glycol standard (M_n_ = 1540 g/mol). Samples for MALDI-TOF MS measurements were prepared as follows: The THF solutions of 2,5-dihydroxy benzoic acid (DHB) (20 mg/mL), samples (10 mg/mL) and sodium trifluoroacetate (5 mg/mL) were mixed in a 10/2/1 (matrix/sample/cationizing agent) volume ratio. A volume of 0.25 μL of the so-prepared MALDI sample solution was deposited onto a metal sample plate and allowed to dry.

#### 3.3.2. Attenuated Total Reflectance Fourier-Transform Infrared (ATR-FT IR)

For recording the attenuated total reflectance (ATR) Fourier-transform infrared (FT IR) spectra, a Perkin Elmer Instruments Spectrum Two FT IR spectrometer equipped with a diamond Universal ATR Sampling Accessory (Perkin Elmer, Waltham, MA, USA) was used, applying four scans and a penetration depth of 6 μm. The average film thickness of the specimens was approximately 0.5 mm. The IR spectra were assessed by the Spectrum ES 5.0 program.

#### 3.3.3. Swelling Experiments

For the determination of the crosslink density by swelling, samples (dimension: 10 mm × 10 mm × ~ 0.3 mm) were swollen in toluene (10 mL) at 29 °C (302 K) in a closed bottle for 24 h according to the procedure presented in [32]. The degree of swelling (Q) and the gel content (G) were calculated by Equations (3) and (4) [32,33].

(3) $$Q=1+\;\frac{\rho_{s}}{\rho_{p}}\left(\frac{m_{2}}{m_{3}}-1\right)$$

(4) $$G\left(\%\right)=\frac{m_{3}}{m_{1}}\cdot 100$$

where ρ_s_ and ρ_p_ are the densities of the solvent (toluene, ρ: 0.8669 g/cm^3^) and SUPU polymers. The masses m_1_, m_2_ and m_3_ are the initial weight, swollen weight and constant weight for the SUPU polymer, respectively.

The crosslink density of the polymers (ν_e_) was calculated using the Flory–Rehner Equation (5) [34]:

(5) $$\nu_{e}=\frac{-\left[\ln\left(1-v_{1}\right){+v}_{1}+\chi \;\cdot {\;v}_{1}{}^{2}\right]}{V_{\mathrm{ms}}\cdot \;\left(v_{1}{}^{1/3}-\frac{v_{1}}{2}\right)}$$

where v_1_ the volume fraction of the polymer and V_ms_ is the molar volume of the solvent.

The solvent–polymer interaction parameter (χ) was determined by Equation (6),

(6) $$\chi =\frac{{\left(\delta_{2}-\delta_{1}\right)}^{2}\cdot {\;V}_{\mathrm{ms}}}{R\;\cdot T}$$

where δ_1_ and δ_2_ stand for the solubility parameter of the solvent and polymer, respectively; R and T are the universal gas constant and the absolute temperature, respectively. For the Hildebrand solubility parameters of toluene and SUPU samples, values of δ_1_ = 18.2 and δ_2_ = 20.5 (MPa)^1/2^ were used, respectively [35]. The molar volume of toluene (V_ms_) and interaction parameter (χ) at 302 K were calculated to be 1.06 × 10^−4^ m^3^/mol and 0.223, respectively.

#### 3.3.4. Scanning Electron Microscopy (SEM)

For the investigation of the morphology of the sucrose containing SUPU samples, scanning electron microscopy (SEM) was employed. SEM images were obtained from the surface of SUPU samples covered with a 30 nm conductive gold layer by means of a Hitachi S-4800 scanning electron microscope (Hitachi, Tokyo, Japan) equipped with a Bruker energy dispersive X-ray spectrometer (Bruker Daltoniks, Bremen, Germany). For microscopic examinations, 1 cm × 1 cm (area 1 cm^2^) samples with an average thickness of 0.5 mm were excised. For the SEM studies, a 15 kV accelerating voltage in the secondary electron mode was used.

#### 3.3.5. Mechanical Test

Tensile test experiments were performed according to the EN ISO 527-1 standard using a computer-controlled Instron 3366 (Instron, Norwood, MA, USA) type tensile testing machine, equipped with a 10 kN load cell. In line with the ASTM D882-12 standard, at least 5 dumbbell specimens were cut (clamped length 60 mm) and loaded at a crosshead speed of 50 mm/min. The measurements were evaluated by means of the Instron Bluehill Universal V 4.05 (2017) software.

#### 3.3.6. Differential Scanning Calorimetry (DSC)

The thermal properties of the sample SUPUs were investigated by means of differential scanning calorimetry (DSC). DSC tests (METTLER TOLEDO, Columbus, OH, USA) were run in DSC 3 power compensation equipment working at a heating rate of 10 °C/min under a protective nitrogen atmosphere. The weight percentage of the crystalline PCD or PCL (C_r_) was determined by Equation (7) [36],

(7) $$C_{r}\left(\%\right)=\frac{\Delta H_{m}}{\chi_{\mathrm{PCD}\;\mathrm{or}\;\mathrm{PCL}}\;{{\Delta H}_{m}}^{0}}\;\cdot 100$$

where ΔH_m_ stands for the heat of fusion of the investigated PU, χ is the weight fraction of PCL in the corresponding SUPU, and ΔH_m_^0^ is the heat of fusion of the pure 100% crystalline PCD (142 kJ/mol) or PCL (139.5 kJ/mol) [37,38].

## 4. Conclusions

Our aim was to synthesize polyurethanes involving sucrose-HDI cooligomer chains. First, the synthesis of sucrose-HDI cooligomer was performed using a 1:1 molear ratio of sucrose and HDI. According to the MALDI-TOF MS measurements, a 4 h reaction was found to be optimal to obtain mostly linear sucrose-HDI cooligomers, while longer reaction times led to the formation of branched oligomers.

The so-obtained sucrose-HDI cooligomers were reacted with the prepolymers synthesized from PCL or PCD and MDI or HDI to yield SUPU networks. The networks were characterized by MALDI-TOF MS, ATR-FT IR, swelling and physical tests. It was found that the unprotected sucrose reacts with HDI without a catalyst, yielding linear sucrose-HDI cooligomers, which were transformed into flexible SUPU networks with isocyanate terminated PCL prepolymers.

The obtained SUPU networks showed improved elasticity and a lower degree of crystallinity comparing to PCL///PCD segments (see the degree of crystallinity of SUPU 1 from 60% to 38% and SUPU 3 from 63% to 8% in Table 3).

## Figures and Tables

**Table 1:** Density and swelling properties at 302 K of SUPUs 1–4 in toluene.

Sample Name	Density (g/cm^3^)	Q	G (%)	v_1_	Crosslink Density (mol/cm^3^)
SUPU 1	1.08	15.7	33.0	0.130	1.2 × 10^−4^
SUPU 2	1.06	8.1	51.3	0.169	2.0 × 10^−4^
SUPU 3	1.01	2.0	96.2	0.439	1.7 × 10^−3^
SUPU 4	1.06	-	-	-	- *

* Dissolved in Toluene.

**Table 2:** Tensile mechanical properties of the samples SUPU 1–4. Designations: E-elastic modulus (Young’s modulus), ε_R_ is the ultimate elongation, and σ_R_ is the stress at break.

Code	E (MPa)	ε_R_ (%)	σ_R_ (MPa)
SUPU 1	147 ± 22	589 ± 67	16.6 ± 2.1
SUPU 2	87.6 ± 18	405 ± 21	6.7 ± 0.2
SUPU 3	16.6 ± 5.6	784 ± 38	6.5 ± 0.9
SUPU 4	49.5 ± 4.3	13 ± 2	1.4 ± 0.3

**Table 3:** Glass temperature (T_g_), melting temperature (T_m_) derived from onset temperature, melting enthalpy (ΔH_m_) and crystallinity values (C_r_) obtained from DSC measurements for PCL segments in SUPUs 1–4 by the analysis of the first heating cycle.

Code	T_g_ (°C)	T_m_ (°C)	ΔH_m_ (J/g)	C_r_ (%)
PCD(2)	-	47	85.3	60
PCL(10)	-	64	87.4	63
SUPU 1	-	56	42.6	38
SUPU 2	-	53	41.3	37
SUPU 3	−44.2	38	4.9	8
SUPU 4	-	47	40.6	64

**Table 4:** The amount of the components used for the syntheses of sucrose-HDI cooligomer and PU-prepolymer.

Sample Name	Sucrose-HDI Cooligomer		PU-Prepolymer			
	Sucrose g/(mmol)	HDI (mmol)	PCL(10) g/(mmol)	PCD(2) g/(mmol)	HDI (mmol)	MDI g/(mmol)
SUPU 1	0.5/(1.5)	1.5	3.65/(0.4)	-	-	0.18/(0.8)
SUPU 2	0.5/(1.5)	1.5	3.65/(0.4)	-	0.8	
SUPU 3	1.5/(4.4)	1.5	-	2.2/(1.1)	-	0.55/(2.4)
SUPU 4	1.5/(4.4)	1.5	-	2.2/(1.1)	2.2	-

**Table 5:** The compositions (mole ratio) of reaction mixtures SUPU 1–4.

Sample Name	Sucrose Oligomer	PU-Prepolymer		PCL/Sucrose
	Sucrose/HDI	Composition	Mole Ratio	
SUPU 1	1/1	PCL(10)-MDI	1/2	1/4
SUPU 2	1/1	PCL(10)-HDI	1/2	1/4
SUPU 3	1/1	PCD(2)-MDI	1/2	1/4
SUPU 4	1/1	PCD(2)-HDI	1/2	1/4

## Data Availability

The data supporting the findings presented in this study are available from the corresponding author on request.

## Supplementary Materials

The following supporting information can be downloaded at: https://www.mdpi.com/article/10.3390/ijms23031444/s1.
