# Supplementary material for: Synthesis of Sucrose-HDI Cooligomers: New Polyols for Novel Polyurethane Networks
Source: Int J Mol Sci. 2022 Jan 27;23(3):1444. doi: 10.3390/ijms23031444 (PMC8836168; doi:10.3390/ijms23031444)
Supplement: Supplementary file 1 [file ijms-23-01444-s001.zip › ijms-1536691-supplementary.pdf]

Synthesis of sucrose-HDI cooligomers: new polyols for novel polyurethane networks

Csilla Lakatos[1], Marcell Árpád Kordován[1,2], Katalin Czifrák[1], Lajos Nagy[1], Bence Vadkerti[1,2], Lajos Daróczi[3], Miklós Zsuga[1] and Sándor Kéki[1*]

[1] Department of Applied Chemistry, University of Debrecen, Egyetem tér 1, H-4032 Debrecen, Hungary;

[2] Doctoral School of Chemistry, University of Debrecen, Egyetem tér 1, H-4032 Debrecen, Hungary

[3] Department of Solid State Physics, University of Debrecen, Bem tér 18/b, H-4026 Debrecen, Hungary

*Correspondence: keki.sandor@science.unideb.hu; Tel.: +36-52-512-900 (ext. 22455)

Table of contents:

Figure S1: MALDI-TOF MS spectra from sucrose-HDI reaction after 144 hours
in linear mode ……………………………………………………………………………....1

Figure S2: IR spectra of sucrose and sucrose-HDI cooligomer……………………………….1

Figure S3: IR spectra of PCD, PCD-HDI prepolymer and SUPU 4…………………………..2

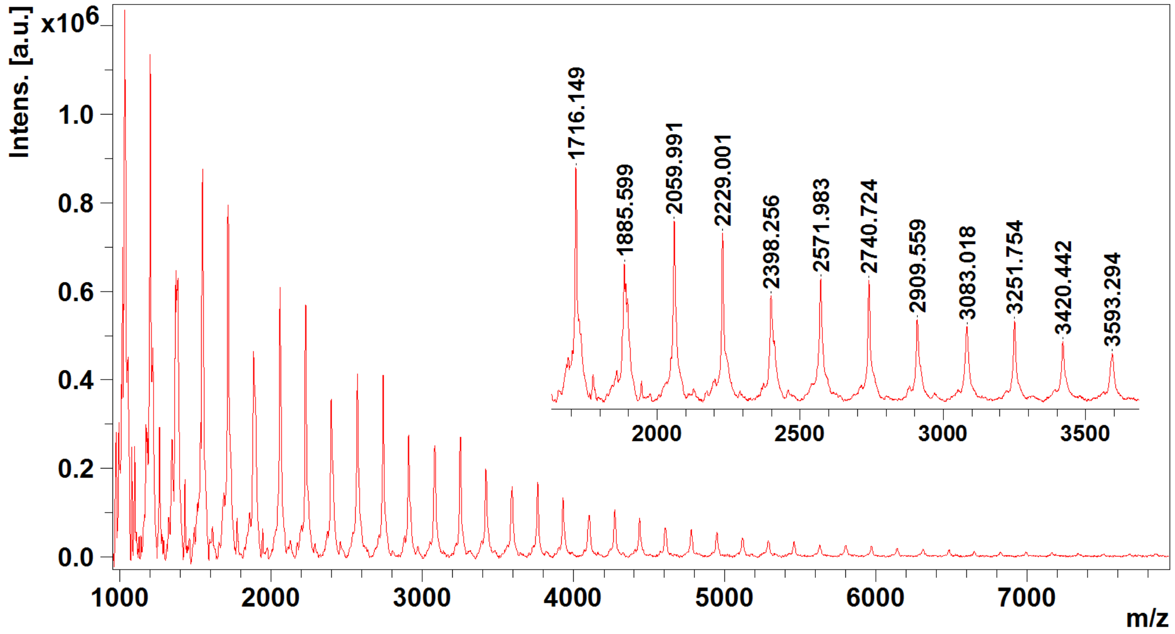

Figure S1: MALDI-TOF MS spectra from sucrose-HDI reaction after 144 hours in linear mode

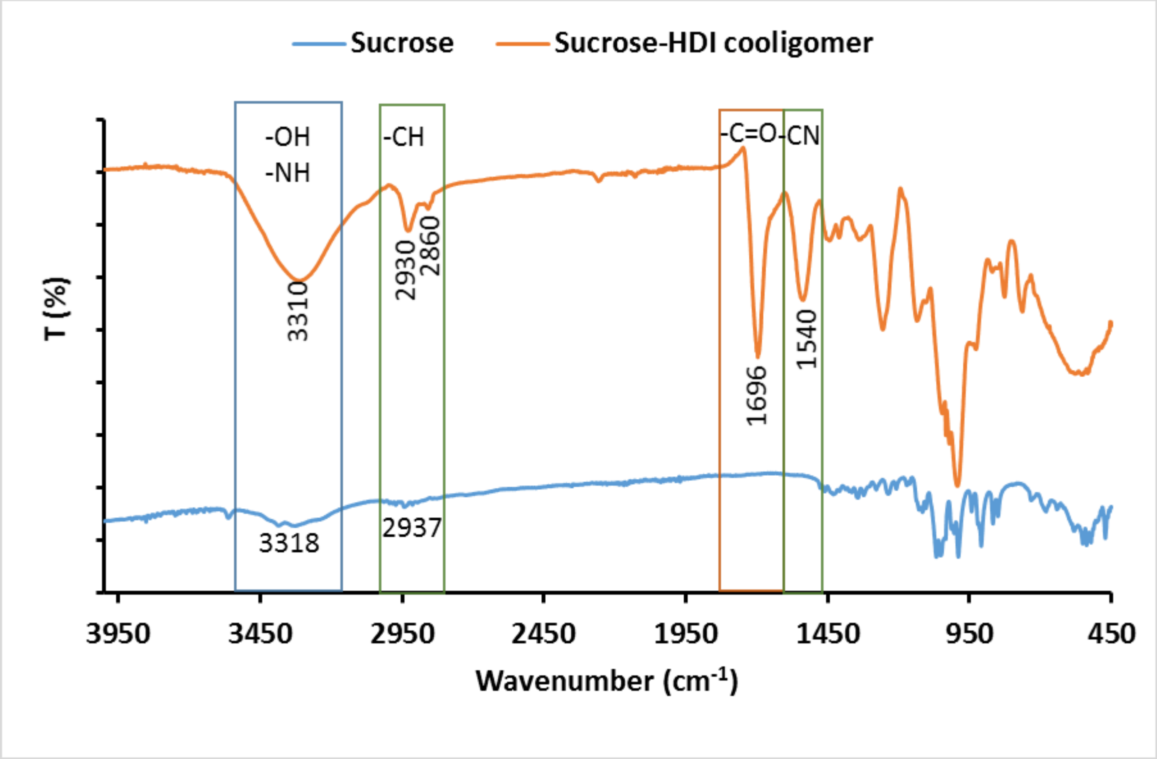

Figure S2: IR spectra of sucrose and sucrose-HDI cooligomer. The appearing –C=O and amide II vibrations in the IR spectrum confirms the formation of the cooligomer.

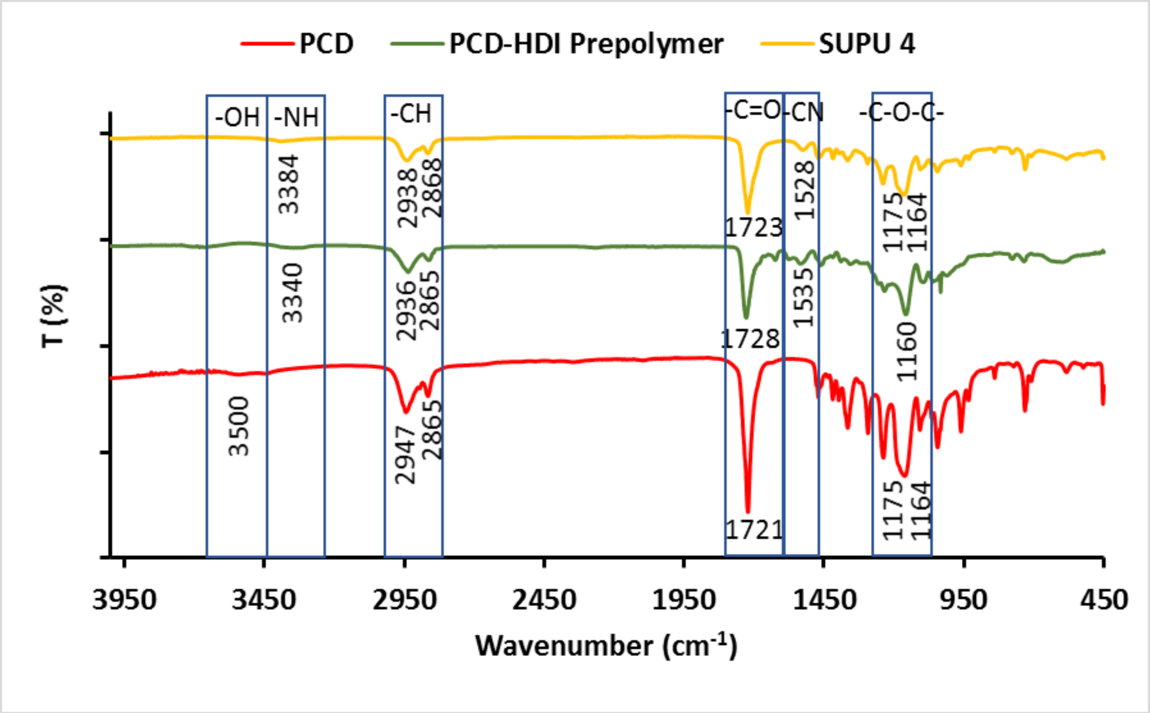

Figure S3: IR spectra of PCD, PCD-HDI prepolymer and SUPU 4. As seen from IR spectra of PCD-HDI prepolymer and SUPU 4 the appearance of –NH streching (between 3384-3340 cm^{-1}) and amide II vibrations (between 1535-1528 cm^{-1}) supports the formation of the desired polymer structure.
